# Continuous central venous oxygen saturation assisted intraoperative hemodynamic management during major abdominal surgery: a randomized, controlled trial

**DOI:** 10.1186/s12871-015-0064-2

**Published:** 2015-06-04

**Authors:** András Mikor, Domonkos Trásy, Márton F Németh, Angelika Osztroluczki, Szilvia Kocsi, Ildikó Kovács, Gábor Demeter, Zsolt Molnár

**Affiliations:** 1 Department of Anaesthesiology and Intensive Therapy, University of Szeged, 6. Semmelweis str., 6725 Szeged, Hungary; 2 Hungarian Defence Forces Medical Center, Budapest, Hungary

**Keywords:** Haemodynamic management, Central venous oxygen saturation, Postoperative complications

## Abstract

**Background:**

Major abdominal surgery is associated with significant risk of morbidity and mortality in the perioperative period. Optimising intraoperative fluid administration may result in improved outcomes. Our aim was to compare the effects of central venous pressure (CVP), and central venous oxygen saturation (ScvO_2_)-assisted fluid therapy on postoperative complications in patients undergoing high risk surgery.

**Methods:**

Patients undergoing elective major abdominal surgery were randomised into control and ScvO_2_ groups. The target level of mean arterial pressure (MAP) was ≥ 60 mmHg in both groups. In cases of MAP < 60 mmHg patients received either a fluid or vasopressor bolus according to the CVP < 8 mmHg in the control group. In the ScvO_2_ group, in addition to the MAP, an ScvO_2_ of <75 % or a >3 % decrease indicated need for intervention, regardless of the actual MAP. Data are presented as mean ± standard deviation or median (interquartile range).

**Results:**

We observed a lower number of patients with complications in the ScvO_2_ group compared to the control group, however it did not reach statistical significance (ScvO_2_ group: 10 *vs.* control group: 19; *p* = 0.07). Patients in the ScvO_2_ group (*n* = 38) received more colloids compared to the control group (*n* = 41) [279(161) *vs.* 107(250) ml/h; *p* < 0.001]. Both groups received similar amounts of crystalloid (1126 ± 471 *vs.* 1049 ± 431 ml/h; *p* = 0.46) and norepinephrine [37(107) *vs.* 18(73) mcg/h; *p* = 0.84]. Despite similar blood loss in both groups, the ScvO_2_ group received more blood transfusions (63 % *vs.* 37 %; *p* = 0.018). More patients in the control group had a postoperative PaO_2_/FiO_2_ < 200 mmHg (23 *vs.* 10, *p* < 0.01). Twenty eight day survival was significantly higher in the ScvO_2_ group (37/38 *vs.* 33/41 *p* = 0.018).

**Conclusion:**

ScvO_2_-assisted intraoperative haemodynamic support provided some benefits, including significantly better postoperative oxygenation and 28 day survival rate, compared to CVP-assisted therapy without a significant effect on postoperative complications during major abdominal surgery.

**Trial registration:**

ClinicalTrials.gov NCT02337010.

## Background

There are an estimated 234 million surgical operations worldwide every year, with significant risk of morbidity and mortality in the perioperative period in patients undergoing major surgery [1]. Following the implementation of safety standards outcomes after anaesthesia have improved, although estimations of perioperative complications and postoperative morbidity are difficult. It has been suggested this may be between 3 and 17 % of cases [2, 3].

Several studies have revealed that inappropriate intraoperative fluid therapy may be responsible for postoperative complications and organ failure. Excessive fluid administration during surgical procedures may lead to more frequent postoperative complications [4, 5], while restrictive fluid therapy may improve outcome after major elective gastrointestinal surgery [6]. On the other hand, fluid restriction may increase the level of hypovolemia and hence hypoperfusion, and thereby increased incidence of postoperative complications [7].

It is well known, that using heart rate (HR), mean arterial pressure (MAP) and central venous pressure (CVP) to assess and guide haemodynamic support may be misleading [8–10].

Advanced haemodynamic monitoring, using cardiac output, stroke volume, stroke volume variation (SVV), pulse pressure variation (PPV) to guide intraoperative fluid therapy has resulted in improved outcomes in several studies [11–14]. Despite the increasing evidence, advanced haemodynamic monitoring has not become routine practice and, in high risk patients, arterial and central venous pressure monitoring remain the most common tools applied in more than 80 % of cases in Europe and in the United States [15]. One of the reasons may be that accurate measurement of cardiac output, SVV and PPV require advanced instrumentation.

Another important factor for haemodynamic stability is the balance between oxygen delivery (DO_2_) and consumption (VO_2_). Unfortunately, detailed haemodynamic evaluation, including DO_2_/VO_2_ balance, for every high risk patient in the operating theatre is not feasible. The most often used bedside parameter to assess the relationship between oxygen supply and consumption is the central venous oxygen saturation (ScvO_2_). Continuous monitoring of the ScvO_2_ is also possible with a device based on fiber-optic technology via a standard central venous catheter. Values measured by this approach have shown good correlation with laboratory values [16]. ScvO_2_ reflects important changes in the DO_2_/VO_2_ relationship, has been found to be useful during high-risk surgery, and low ScvO_2_ is associated with increased postoperative complications [17, 18]. Despite these theoretical advantages, ScvO_2_ is only used in 12–30 % of high risk surgical patients [15]. In clinical routine, MAP and CVP are the most frequently applied monitoring tools (75–95 %) during high risk surgery [15], despite convincing evidence that neither can predict fluid responsiveness [8–10].

Therefore, the aim of the current study was to compare the effects of ScvO_2_ assisted intraoperative haemodynamic support to the routinely used MAP-CVP approach on postoperative complications in high risk surgical patients.

## Methods

### Patients

Following Regional Ethics Committee approval (details are summarised below) and obtaining written informed consent, all patients undergoing the following elective major abdominal surgeries, including oesophagectomy, total gastrectomy, radical cystectomy, aorto-bifemoral bypass or elective repair of abdominal aortic aneurysm, were enrolled into our prospective study. After surgery all patients were admitted to our intensive care unit (ICU) in Department of Anaesthesiology and Intensive Therapy, University of Szeged, Hungary. Exclusion criteria were pre-existing chronic organ insufficiency as determined by the Acute Physiology and Chronic Health Evaluation (APACHE) II scoring system, New York Heart Association Class IV, chronic hypoxia or hypercapnia, chronic renal failure requiring renal replacement therapy, biopsy proven cirrhosis or portal hypertension and immunodeficiency [19]. Furthermore, in cases of preoperative anaemia (haemoglobin < 100 g/L), coagulation abnormality, and patients with chronic use of corticosteriods and non-steroid anti-inflammatory drugs were also excluded. Patients requiring an operation due to malignant disease where the tumour then proved to be inoperable were also excluded.

Patients were randomly allocated by envelope randomisation in a block-of-ten fashion into control, or ScvO_2_ groups.

### Anaesthesia and monitoring

All patients received routine anaesthetic management, premedication with oral benzodiazepine, induction with propofol (1–2 mg/kg), muscle relaxation with rocuronium (0.6 mg/kg) and analgesia with intravenous fentanyl (0.7–1 mcg/kg/dose). If an epidural catheter was inserted, it was tested with 60 mg lignocaine but during the operation only intravenous analgesia was used to prevent hypotension caused by epidural analgesia. Anaesthesia was maintained with sevoflurane (minimum alveolar concentration (MAC): 1.0–1.2). After endotracheal intubation, arterial and internal jugular central venous catheters were inserted. During the surgical procedures haemodynamic parameters (heart rate, invasive blood pressure, CVP), oxygen saturation (SaO_2_), end-tidal CO_2_ tension, respiratory gases, and urine output were monitored. Ventilation was maintained with a peep end-expiratory pressure of 4 cmH_2_O, tidal volume 6–8 ml/kg and fraction of inspired oxygen (FiO_2_) 0.4–0.5 to maintain SaO_2_ > 94 % and end-tidal CO_2_ tension of 35–40 mmHg. In all patients lactated Ringer’s solution (10–15 ml/kg/h) was infused as the baseline volume replacement. Arterial and central venous blood samples were taken hourly for blood gas analysis. The amount of crystalloid and colloid infusion administered, the demand and dose of vasopressor/inotropic support and blood transfusions were all recorded at the end of surgery.

### Measurement of ScvO_2_

Central venous saturation was continuously monitored in the ScvO_2_ group by using a CeVOX monitor (Pulsion Medical Systems, Munich, Germany). The CeVOX probe (PV2022-37; Pulsion Medical Systems, Munich, Germany) was inserted into the internal jugular central venous catheter as described in the manufacturer’s users manual. The position of the central venous catheter in the superior vena cava was confirmed by chest X-ray postoperatively. The system was calibrated *in vivo* for ScvO_2_ measurements by laboratory co-oximeter (Cobas b 221, Roche Ltd, Basel, Switzerland). Calibration, if necessary, was repeated at least hourly during the surgical procedure. In the control group the level of central venous saturation was measured hourly by laboratory co-oximeter.

### Interventions and protocol

The anaesthetist responsible for the patient was blinded to the ScvO_2_ in the control group and to the CVP in the ScvO_2_ group. Regarding interventions in general, if hypovolaemia was suspected (see below) fluid bolus was given in the form of 250 ml hydroxyethyl starch solution (HES, 6 % hydroxyethyl starch 130/0.4 in 0.9 % sodium chloride, Voluven, Fresenius Kabi, Germany) over 15 min. If hypovolaemia was unlikely, but hypotension was present this was treated with a vasopressor (10 mcg bolus or continuous infusion of norepinephrine).

In the control group cases of hypotension (as defined by MAP < 60 mmHg) were treated with a fluid bolus if the CVP < 8 mmHg, and norepinephrine if the CVP ≥ 8 mmHg, reflecting the clinical routine. These target values are also recommended in several (albeit not intra-operative), guidelines [20, 21].

**Fig. 1:**
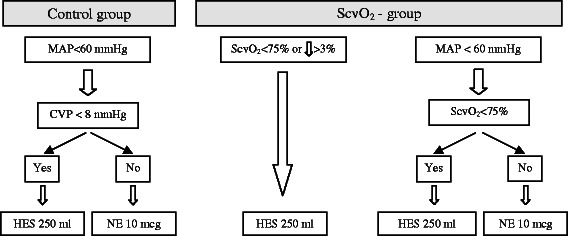
Flowchart of the study design. MAP: mean arterial pressure, CVP: central venous pressure, ScvO_2_: central venous oxygen saturation, HES: hydroxyethyl starch, NE: norepinephrine

In the ScvO_2_ group, hypotension (MAP < 60 mmHg) was considered primarily due to hypovolaemia if the ScvO_2_ < 75 %, and patients received a fluid bolus. If the ScvO_2_ ≥ 75 %, it was assumed that hypotension was primarily due to vasodilatation caused by general anaesthesia, and norepinephrine was administrated. In addition to low MAP there was also another trigger for intervention in this group: if ScvO_2_ dropped below 75 % or there was a sudden decrease by more than >3 %, patients received a fluid bolus regardless of the MAP. The main steps of the protocol are summarised in Fig. 1. The effect of the administered fluid bolus was reassessed in every 15 min. It is important to note that in cases of persistent hypotension treated by the fluid bolus as per the study protocol, anaesthetists were allowed to administer norepinephrine boluses in both groups and the amount given was recorded and added to the total dose calculated at the end of surgery. Intraoperative transfusion was indicated if the haemoglobin level was below 80 g/l as determined by blood gas analysis. Intraoperative blood recovery techniques were not used.

During the operation arterial and central venous blood gas analysis were done hourly. Blood samples for laboratory assessments such as kidney function, liver function, blood count and inflammatory parameters such as procalcitonin (PCT) and C-reactive protein (CRP) were taken before the operation, on arrival to the ICU and 24, 48 h later. Arterial and central venous blood gas analyses were also performed at these time points.

### Statistics

All data are presented as mean ± SD or median (interquartile range) as indicated by data distribution tested by the Shapiro-Wilk test. Independent samples *T*-test or Mann–Whitney *U* test were used to compare the data between the two groups depending on data distribution in each measurement. To evaluate changes in the measured parameters over time within the groups, two-way analysis of variance (ANOVA) was used. To assess the difference between categorical data we used Pearson’s chi-squared test.

The main outcome parameter was the incidence of postoperative complications on the first and second postoperative day. We calculated the number of patients observed with pulmonary, circulation, abdominal, renal, infectious or surgical complications based on a previous study by Mayer at al. [22]. Following completion of the study, respiratory complications and acute kidney injury were analysed post hoc. Pulmonary function was assessed by using the ratio of arterial partial oxygen tension and the fraction of inspired oxygen (PaO_2_/FiO_2_) according to the Berlin definition of acute respiratory distress syndrome [23]. To assess the severity of kidney disease we used the Kidney Disease Improving Global Outcome (KDIGO) acute kidney injury definition [24]. Secondary end points were the difference in intraoperative fluid and vasopressor requirements. Based on the results of a previous study on a similar patient population [22], it was found that in the control group the incidence of organ dysfunction was 50 %, whereas in the goal directed therapy group it was only 20 % (*i.e.* the difference was 30 %). Therefore, to have 80 % power if the *p* < 0.05 with Pearson’s chi-squared test, the required number of patients should be a minimum of 40 per group. For statistical analysis the Statistical Package for Social Sciences (SPSS version 20, IBM Corporation, Armonk, NY, United States) software for Windows was used. Statistical significance was considered at *p* < 0.05.

### Ethics

Ethical approval for this study (2618 – 2/2010.) was provided by the Regional Ethical Committee of University of Szeged, Albert Szent-Györgyi Health Center, Szeged, Hungary (Chairperson: Prof. Tibor Wittmann) in 2010.

## Results

**Fig. 2:**
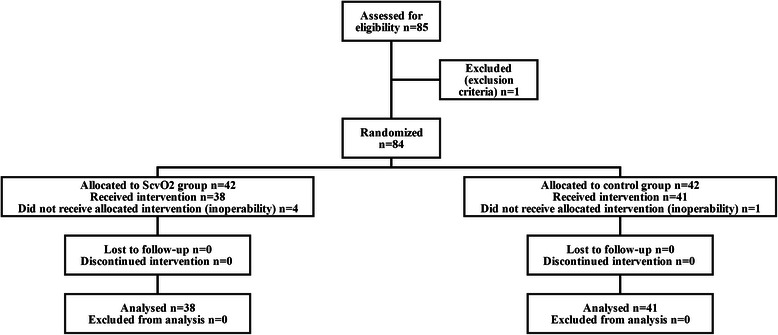
CONSORT flow diagram of the study

Eighty five patients met the inclusion criteria between 2011 and 2013. One patient was excluded due to chronic renal failure hence 42 patients were randomized to each group. Four patients in the ScvO_2_ group and 1 patient in the control group had to be withdrawn from the study due to the inoperability of the tumour (Fig. 2). There were no significant differences between the two groups regarding demographics and clinical characteristics. Five patients in the control group were not extubated at the end of the surgery, 4 of whom were extubated on the first postoperative day and one patient was ventilated for 11 days. In the ScvO_2_ group, all patients were extubated at the end of surgery apart from 2 patients who were extubated on the first postoperative day and 1 patient who was ventilated for 3 days. Following extubation all patients received oxygen supplementation via a 28 % or 40 % Venturi face mask to maintain SaO_2_ > 94 %. Two patients died in the ICU in the control group with 28 days survival also significantly lower in this group (Table 1).

**Table 1:** Demography of the patients. Data are shown as mean ± SD or median (interquartile)

	ScvO_2_ (*n* = 38)	Control (*n* = 41)	*p*
Age (years)	62 ± 8	62 ± 8	0.95
Sex (M/F)	28/10	29/12	0.77
APACHE II	12 ± 4	11 ± 5	0.37
ICU LOS (days)	3 (2)	3 (2)	0.663
Length of operation (min)	247 ± 82	254 ± 45	0.76
Oesophagectomy (number of patients)	4	2	
Total gastrectomy (number of patients)	3	0	
Cystectomy (number of patients)	22	29	
Aortobifemoral bypass (number of patients)	5	7	
Aortic aneurysm (number of patients)	4	3	
ICU survival (S/NS)	38/0	39/2	0.17
28 day survival (S/NS)	37/1	33/8	0.018*

*: p<0.05

**Fig. 3:**
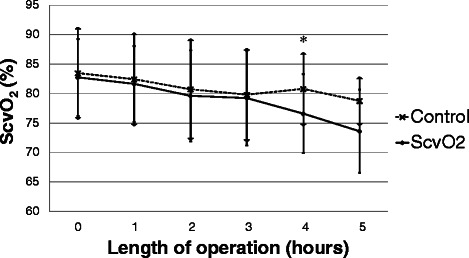
Changes in central venous saturation (ScvO_2_) during the operation. Data are shown as mean and standard deviation

**Fig. 4:**
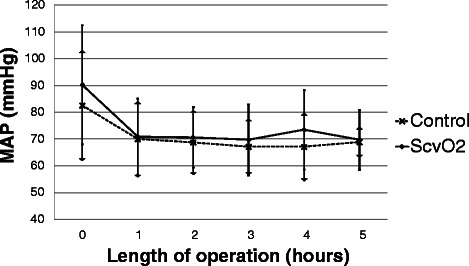
Changes in mean arterial pressure (MAP) during the operation. Data are shown as mean and standard deviation

**Fig. 5:**
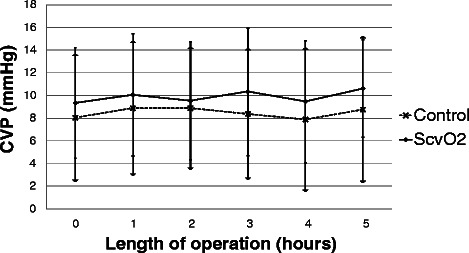
Changes in central venous pressure (CVP) during the operation. Data are shown as mean and standard deviation

**Fig. 6:**
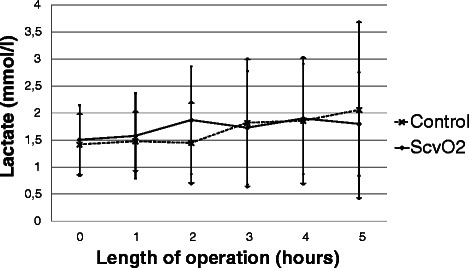
Changes in lactate level during the operation. Data are shown as mean and standard deviation

There was no significant difference in ScvO_2_ between the two groups at baseline. During the operation there was a decrease in ScvO_2_ in the ScvO_2_ group while it remained almost unchanged in the control group, reaching a significant difference between the two groups four hours after the start of the operation (Fig. 3). The target MAP was achieved in most cases with no difference between the groups (Fig. 4). Regarding the CVP there was no significant difference between the two groups throughout the operation (Fig. 5). Measurement of the urine output during the operation was complicated in 33 patients who underwent radical cystectomy. However, in cases where exact measurement was possible, hourly urine output showed a significant difference between the two groups: ScvO_2_ group (*n* = 23): 165 ± 98 ml/h *vs.* controls (*n* = 23): 109 ± 92 ml/h, *p* = 0.023. Although less patients had at least one hypotensive episode during surgery in the ScvO_2_ group (17 *vs.* 25 in the control group), this difference was not statistically significant (*p* = 0.18). Patients received more colloid intraoperatively in the ScvO_2_ group, while the amount of crystalloid infusion administered was similar in both groups. The number of patients who received an intraoperative blood transfusion was also significantly higher in the ScvO_2_ group, although intraoperative blood loss was similar in both groups (Table 2). The haemoglobin levels at the start (ScvO_2_ group: 108 ± 19 g/l *vs.* control: 109 ± 22 g/l) and the end of the operation showed no significant difference (ScvO_2_ group: 94 ± 14 g/l *vs.* control: 97 ± 17 g/l). The lactate levels were normal in both groups during the whole operation without any significant difference or change (Fig. 6). There was no difference between the two groups in the number of patients with vasopressor support and their vasopressor demand during the operation (Table 2).

**Table 2:** Intraoperative interventions. Data are shown as mean ± SD or median (interquartile)

	ScvO_2_ (*n* = 38)	Control (*n* = 41)	*p*
Crystalloid (ml/h)	1126 ± 471	1049 ± 431	0.46
Colloid (ml/h)	279 (161)	107 (250)	<0.001*
Number of patients needing vasopressor	11	15	0.47
Dose of vasopressor (mcg/h)	37 (107)	18 (73)	0.84
Number of patients receiving blood transfusion	24	15	0.02*
Blood loss during the operation (ml)	973 ± 473	983 ± 574	0.99

*: p<0.05

Regarding postoperative complications, there were more patients with complications in the control group but it did not reach statistical significance. However, pulmonary complications as determined by the PaO_2_/FiO_2_ ratio were significantly higher on the first and second postoperative day in the control group (Table 3).

**Table 3:** Postoperative complications within 48 h after the operation. Data are shown as number of patients with each complication. KDIGO: Kidney Disease Improving Global Outcomes staging

		ScvO_2_ (*n* = 38)	Control (*n* = 41)	*p*
Infection	Respiratory	0	1	0.33
	Abdominal	2	2	0.94
	Urinary tract	0	1	0.33
	Wound	0	0	-
Mechanical ventilation > 24 h		1	5	0.11
Circulation	Cardiac decompensation	0	0	-
	Arrhythmia	1	4	0.19
	Vasopressor need	9	14	0.31
	Acute myocardial infarction	0	0	-
	Stroke	0	0	-
Abdominal	Constipation	2	3	0.71
	Upper gastrointestinal bleeding	0	1	0.33
	Re-operation	1	2	0.60
Urine output < 500 ml/24 h or haemodialysis		1	3	0.34
Postoperative surgical bleeding		1	1	0.96
Perioperative deaths		0	1	0.33
Number of patients with complications		10	19	0.07
PaO_2_/FiO_2_	>300 Hgmm	4	3	0.62
	200–300 Hgmm	24	15	0.02*
	100–200 Hgmm	10	22	0.01*
	<100 Hgmm	0	1	0.52
Acute kidney injury	no injury	27	29	0.59
	KDIGO 1	7	10	0.36
	KDIGO 2	3	1	0.28
	KDIGO 3	1	1	0.73

*: p<0.05

There was no difference regarding the dose of fentanyl used during the operation (ScvO_2_: 179 [70] mcg/h *vs.* control: 167 [77] mcg/h, *p* = 0.06). The MAC of sevoflurane remained between 1.0 and 1.2 during the whole operation for both groups with no significant difference.

There were no significant differences in any of the investigated inflammatory markers (CRP, leucocyte count, fever, microalbuminuria – data not shown) throughout the perioperative period. PCT also showed almost identical kinetics and absolute values in the two groups at t_0–24–48_ (ScvO_2_: 0.06 [0.00] - 0.66 [1.21] - 0.45 [0.98]; controls: 0.06 [0.01] - 0.53 [1.4] - 0.42 [1.03] ng/ml, respectively).

## Discussion

In this prospective randomised study we found that ScvO_2_ and MAP based intraoperative haemodynamic management resulted in more intraoperative interventions, better intraoperative diuresis and less pulmonary dysfunction in the postoperative period compared to a MAP and CVP guided therapy, however the overall complication rate was not reduced significantly.

### ScvO_2_ during intraoperative haemodynamic management

It has been shown that ScvO_2_ is a reliable parameter to assess the balance between oxygen supply and demand in critically ill patients [20, 25, 26]. Although controversy still exists about the interpretation of ScvO_2_, it is universally accepted that “low” values suggest a global oxygen debt [26] and subsequently a Collaborative Study Group has warranted clinical trials be performed with goal-directed therapy using ScvO_2_ as a target in high-risk surgical patients [18].

In one of the first studies on this subject it was found that reduced ScvO_2_ in the postoperative period is related to increased post-operative complications. The best cut-off value of ScvO_2_ for predicting complications was found to be 64.4 % in the early post-operative period [17]. However, the “target” or in other words “normal” intraoperative ScvO_2_ value remains uncertain. Theoretically ScvO_2_ should be “higher” than the physiological value determined in awake subjects or found in patients in ICU, due to the reduced oxygen demand/consumption during general anaesthesia. In a recent study in which pre- and postoperative ScvO_2_ values were investigated in patients undergoing major abdominal surgery, the critical value was suggested to be 73 % [18]. There is also data that keeping the oxygen extraction ratio (calculated from the arterial and central venous oxygen saturation) below 27 % resulted in less postoperative organ dysfunction and reduced hospital stay in high-risk surgical patients [27]. In a recent observational study in surgical patients, even higher levels of ScvO_2_ have been reported (84.7 ± 8.3 %) [28]. We had similar findings in a previous pilot study, in which the median ScvO_2_ was 81 % for the whole sample [29]. Therefore, in the current study we decided to use an interventional threshold of ScvO_2_ ≤ 75 % or a decrease of >3 %, and observed more therapeutic interventions compared to the MAP and CVP guided control group: patients received more fluid and blood transfusions. Any decrease in DO_2_ might have been recognised earlier by ScvO_2_ than CVP and resulted in more frequent interventions, similar to the results of Rivers *et al.*, who (although in septic patients) also found that the patients assigned to early goal-directed therapy received significantly more fluid, more red-cell transfusions and inotropic support in the initial phase of resuscitation [20]. As there was no difference between the groups in the haemoglobin levels at the start and at the end of the operation, and the intraoperative blood loss was similar in both groups, the increased use of fluid in the ScvO2 group may had caused dilutional anaemia and the need for more frequent transfusion in this group. These interventions possibly resulted in better tissue perfusion and oxygen delivery, also shown by the significantly better intraoperative diuresis which might have led to better outcomes. Indeed, it has been shown that there is strong relationship between ScvO_2_ and anaemia causing an altered VO_2_/DO_2_ balance [30].

### Fluid intake and outcome

In the ScvO_2_ group patients received more colloid boluses. This is similar to a recent paper by Goepfert *et al.*, in which goal-directed therapy in patients undergoing cardiac surgery, using stroke volume variation and optimised global end diastolic volume index, resulted in significantly more colloid administration both intraoperatively and in the ICU alike, and was accompanied by better outcomes [31]. In our study the number of patients with complications was lower in the ScvO_2_ group who had higher fluid intake during the operation, although the difference was not significant.

Despite the increased fluid administration and transfusion, gas exchange was not affected as indicated by the PaO_2_/FiO_2_ ratio, which was actually higher in the ScvO2 group. We couldn’t identify any early adverse effects from the use of colloid solution as indicated by the renal function tests. Although there was significantly higher 28 day survival in the ScvO_2_ group, but the study wasn’t powered to measure the effect on survival, hence the sample size is too small to draw any conclusion regarding postoperative mortality.

Regarding intraoperative fluid management, there is large body of evidence that “restrictive” fluid strategy during major surgery is superior to “liberal” protocols [32, 33]. This is certainly true when only basic monitoring (blood pressure, heart rate, urine output) is applied. However, whenever advanced haemodynamic targets are used, treatment can be individualised, in other words tailored to the patients’ actual need rather than simply just treating protocol based numbers (MAP or CVP), which may be beneficial for some, but may harm others [34]. There is mounting evidence that dynamic physiological indices based approaches are more beneficial than conventional treatments for patients undergoing high risk surgery [31, 35]. These are also in accordance with the findings of the recent OPTIMISE trial [36], which although could not show any significant reduction in the primary outcome (complication rate at 30 days) in the cardiac output guided group, there was a measurable treatment effect, and at 180 days there was a non-significant reduction in mortality.

### CVP *vs.* ScvO_2_ as therapeutic targets

It has been shown that static preload parameters, including CVP, have limited clinical value in guiding heaemodynamic support and may also be inadequate for predicting fluid responsiveness [10, 37]. In our study there was no significant difference at any time point either between, or within groups for CVP, while ScvO_2_ did change and reached a significant difference between the groups over time. During anaesthesia oxygen consumption is lower than while awake, and both oxygen uptake and demand are more-or-less steady. Therefore, it is reasonable to assume that changes in ScvO_2_ mainly reflected changes in cardiac output and oxygen supply. It has also been shown that there is poor relationship between ventricular filling pressure and ventricular volume, hence CVP is a very crude measure of haemodynamic changes. This relationship could further be disturbed by diastolic dysfunction and altered ventricular compliance [38]. Despite all these data, CVP measurement is still more widely used compared to ScvO_2_ in the intraoperative setting [15].

There was a non-significant gradual decrease in ScvO_2_ in both groups towards the end of surgery, reaching the targeted 75 % in the ScvO2 group. There was no significant difference between the groups initially, but after 4 h ScvO_2_ remained significantly higher in the control group. Whilst there is general consensus that low venous oxygen saturations are an important warning sign for the inadequacy of oxygen delivery [39], high values are more difficult to interpret. High values may mean reduced demand, but may also mean inadequate uptake [40, 41]. Although we cannot prove it, we cannot exclude that the high ScvO_2_ values in the control group may have been the result of inadequate fluid loading causing reduced oxygen uptake.

### Limitations of the study

Although we did perform a power analysis to determine the sample size, this was still a relatively small single centre study. As a result the largest proportion of patients consisted of those who underwent radical cystectomy, which may hinder the application of the results for all types of major surgery. Furthermore, neither cardiac output, nor pulse pressure or stroke volume variations were monitored for more precise haemodynamic evaluation. We commenced this study before we had the results of one of our recent multicentre studies on pulse pressure variation/cardiac index/MAP guided intraoperative management [35]. On the other hand, continuous monitoring of dynamic parameters such as SVV or PPV are not the part of the routine haemodynamic assessment and management during these operations. However, regarding these procedures, introduction of a central venous line is part of the routine approach, therefore the measurement of ScvO_2_ provided an easily obtainable alternative for optimising intraoperative haemodynamics. Finally, depth of anaesthesia measurement with bispectral index monitoring was not applied although it is well known that awareness can have a significant effect on hemodynamic responses. However, as the anaesthetic protocols were the same in both groups, and as the MAC values and opioid consumption were also similar it is felt that this may not impact on the results.

## Conclusions

In the current study, using ScvO_2_ as a haemodynamic end-point in addition to MAP, resulted in more intraoperative fluid administration and transfusion during major abdominal surgery. Based on our results, as the insertion of a central venous line is part of the routine management of these surgical procedures, instead of advanced haemodynamic monitoring, ScvO_2_ assisted intraoperative haemodynamic management may be a useful alternative and may also lead to improved outcomes. This study also supports our previous assumption that if ScvO_2_ is used during general anaesthesia, higher levels should be considered as a target value than in the critical care setting.

## Abbreviations

APACHE: Acute physiology and chronic health evaluation
CRP: C-reactive protein
CVP: Central venous pressure
DO_2_: Oxygen delivery
FiO_2_: Fraction of inspired oxygen
HES: Hydroxyethyl starch
HR: Heart rate
KDIGO: Kidney disease improving global outcome
MAC: Minimum alveolar concentration
MAP: Mean arterial pressure
PCT: Procalcitonin
PPV: Pulse pressure variation
SaO_2_: Arterial oxygen saturation
ScvO_2_: Central venous saturation
SVV: Stroke volume variation
VO_2_: Oxygen consumption
